# Airway Emergency Drill: An In Situ Simulation to Increase Staff Preparedness for Airway Bleeding

**DOI:** 10.1002/lary.32458

**Published:** 2025-08-02

**Authors:** Kyle Polen, Caitlin Pickeral, Kara R. Silberthau, Tiffany N. Chao

**Affiliations:** ^1^ Perelman School of Medicine at the University of Pennsylvania Philadelphia Pennsylvania USA; ^2^ Department of Otorhinolaryngology—Head and Neck Surgery University of Pennsylvania Philadelphia Pennsylvania USA

**Keywords:** airway emergency, multidisciplinary, quality and safety, simulation

## Abstract

**Objective:**

In situ simulation offers a valuable opportunity for teams to refine emergency response skills within their actual clinical environment. To evaluate the efficacy and utility of this model, we implemented an in situ emergency airway drill simulation on the otolaryngology inpatient floor at a quaternary care hospital and assessed participant‐reported outcomes.

**Methods:**

Twelve in situ airway emergency simulations were conducted over 3 years, involving 63 Airway Rapid Response Team (ARRT) otolaryngologists, anesthesiologists, respiratory therapists, surgical intensivists, nurses, and pharmacists. Emergency scenarios simulated real‐world airway crises. Surveys assessed provider confidence, anxiety reduction, subjective value of the experience, and perceived impact on patient safety. Confidence ratings were collected pre‐ and post‐simulation in a subset of 34 participants and analyzed via paired *t*‐test. Qualitative feedback from debriefing sessions was thematically assessed.

**Results:**

Post‐simulation surveys demonstrated 72% of participants reported increased confidence in airway emergencies, and 86% reported reduced anxiety compared to before the simulation. It was also found that 93% of participants valued the experience, and 100% agreed that the in situ drill will positively impact patient safety. Confidence increased significantly among participants who completed pre‐ and post‐simulation assessments (5.8–7.6 on a 10‐point scale; *p* < 0.0001). Qualitative themes highlighted increased familiarity with airway equipment and improved interdisciplinary communication, with 56% of participants independently citing these as main takeaways.

**Conclusion:**

In situ airway emergency drills are an effective tool for training multidisciplinary teams, as they improve provider confidence, familiarity with equipment, and interprofessional collaboration in preparation for high‐stakes airway emergencies.

**Level of Evidence:**

N/A.

## Graphical abstract

This study demonstrates the effectiveness of an in situ simulation format in enhancing preparedness for high‐stakes airway emergencies. The approach significantly increased provider confidence, reduced anxiety, improved interdisciplinary communication, and enhanced familiarity with equipment among providers within the multidisciplinary Airway Rapid Response Team.

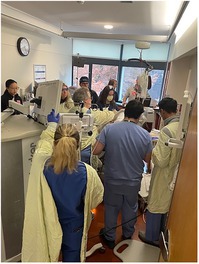

## 1 Introduction

In situ simulation is increasingly gaining recognition as a valuable way to prepare healthcare teams for emergency situations [1]. Unlike traditional simulation‐based education conducted in separate training facilities, in situ simulation takes place in the actual clinical environment where participants work. This allows for real‐time assessment of team dynamics, equipment accessibility, and system‐based challenges [2]. The in situ training approach has been widely adopted in the field of emergency medicine to promote preparedness and patient safety [3], and given the frequent overlap in airway management, otolaryngology teams may benefit from similarly structured training. However, there is currently a paucity of data on its implementation in this specialty.

Patients undergoing otolaryngologic procedures are at heightened risk for airway emergencies, necessitating rapid intervention, readily available equipment, and a well‐coordinated response from skilled personnel [4]. Many hospitals have dedicated otolaryngology nursing units and Airway Rapid Response Teams (ARRTs) or Difficult Airway Response Teams (DART) designed to manage such critical events [5, 6, 7]. Despite these existing structures, standardized collaborative training protocols and opportunities to work together prior to emergency events are often lacking.

To address this gap, a series of in situ airway emergency drills were developed through a multidisciplinary collaboration alongside leadership from the healthcare system's simulation department. While most airway emergency simulations described in the literature involve otolaryngology residents in simulation centers [8, 9], this in situ airway emergency drill is novel in that it takes place on the otolaryngology inpatient floor, incorporating the multidisciplinary team that routinely responds to airway emergencies. This study aims to evaluate the efficacy and impact of a series of in situ airway emergency drill simulations for the ARRT in a quaternary care hospital, with the ultimate goal of enhancing interdisciplinary teamwork, patient safety, and clinical outcomes. We hypothesize that implementing in situ airway emergency drill simulations will increase provider confidence, decrease anxiety toward responding to emergencies, be deemed valuable by participants, and be perceived as beneficial to patient safety.

## 2 Materials and Methods

Over a 3‐year period, 12 airway emergency simulations were conducted with 63 participants from the ARRT. Simulations were conducted in four sessions: the first two simulations each included a single airway emergency drill, whereas the latter two sessions incorporated five drills each, integrating feedback from the earlier simulations. These sessions were developed in collaboration with the Penn Educational Leadership Simulation Program and the Penn Medicine Department of Otorhinolaryngology—Head and Neck Surgery to ensure educational rigor and scenario relevance. The ARRT included otolaryngology faculty, residents, advanced practice providers, and nurses, as well as providers from anesthesiology, the surgical intensive care unit, respiratory therapy, and pharmacy.

**FIGURE 1:**
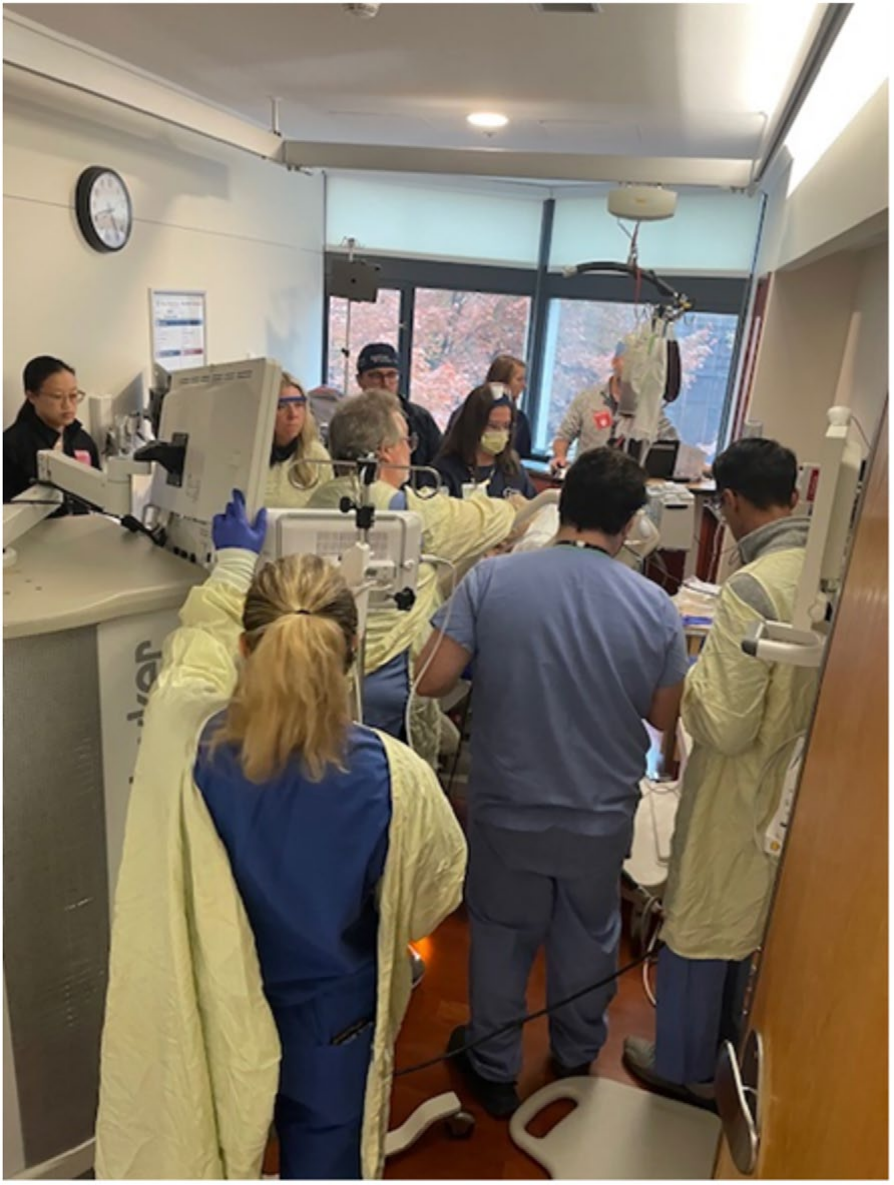
In situ simulation in progress. Photograph of the emergency airway drill in progress with the multidisciplinary team. [Color figure can be viewed in the online issue, which is available at www.laryngoscope.com.]

The emergency scenarios were designed to simulate real‐world airway emergencies, particularly cases involving airway bleeding. Participants were not briefed on the nature of the simulation beforehand. Team members were summoned to a patient room, and the simulation would begin after a 5‐min introduction of the scenario (Figure 1). Each session lasted approximately 20 min to reflect typical emergency response times.

To evaluate the effectiveness of the simulations, participants reported their baseline level of confidence in responding to an Airway Rapid Response on a numerical scale before the exercise, post‐simulation questionnaires were administered (Table 1), and a formal multidisciplinary debriefing session was conducted. Changes in confidence levels were assessed using a paired *t*‐test with data from the two simulation sessions (*n* = 34) in which both pre‐ and post‐intervention confidence ratings were collected. Qualitative and quantitative assessments focused on providers' perception of the experience, team dynamics, and familiarity with airway management equipment. Descriptive statistics were used to quantify participants' reports. A mixed‐methods approach was also utilized to analyze qualitative feedback gathered from debriefing discussions and open‐ended participant questionnaire responses. De‐identified participant data was analyzed using Microsoft Excel (Microsoft Corporation) and GraphPad Prism (GraphPad Software Inc.), and all simulations were video recorded with participants' consent for educational review and future trainings. The project was reviewed and determined to qualify as quality improvement by the University of Pennsylvania's Institutional Review Board.

**TABLE 1:** Participant questionnaire.

Question	Response options
1. After today's simulation experience, how would you rate your confidence in responding to an Airway Rapid Response of an airway bleeding patient?	0–10
2. After participating in today's simulation, I will have a decreased level of anxiety when responding to future Airway Rapid Responses.	Yes/no
3. This type of simulation in the inpatient setting was a valuable experience and should be continued.	Yes/no
4. This type of simulation would have a positive impact on patient care and safety.	Yes/no

*Note*: Survey administered to participants following the in situ simulation.

## 3 Results

**FIGURE 2:**
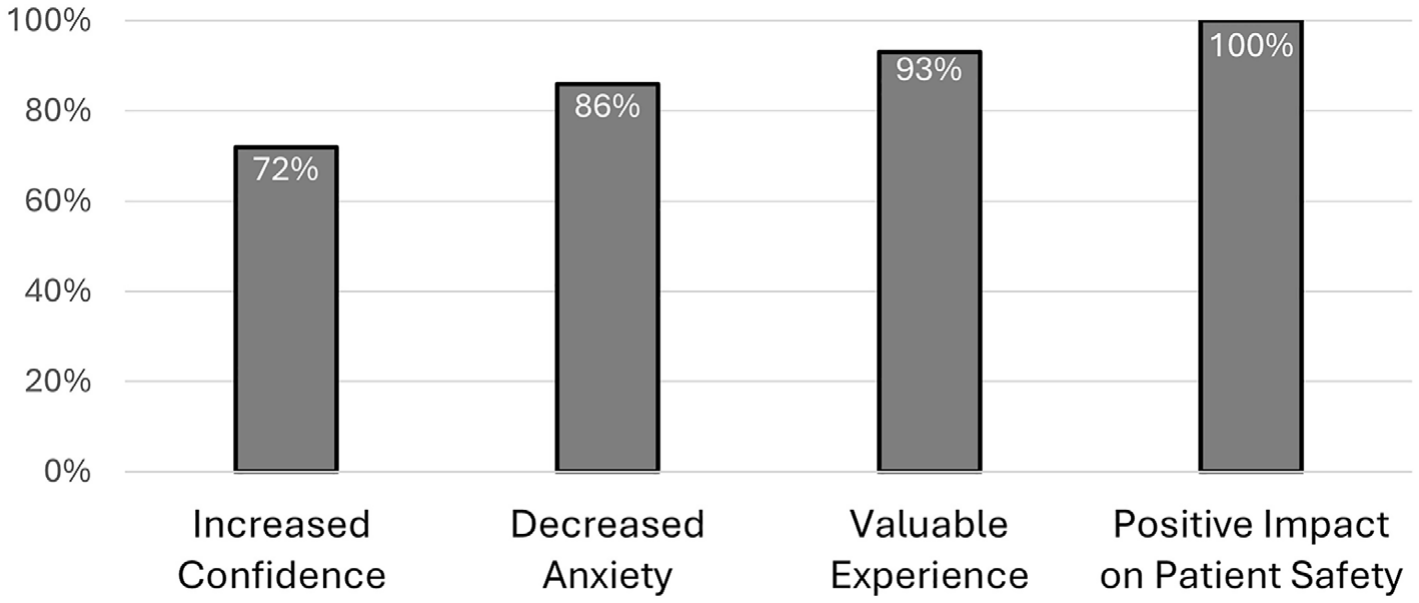
Participant reported outcomes. Results of the post‐simulation survey, represented as the proportion of respondents who had an increase in their self‐reported confidence score and those who indicated “yes” to corresponding binary questions (*n* = 63).

**FIGURE 3:**
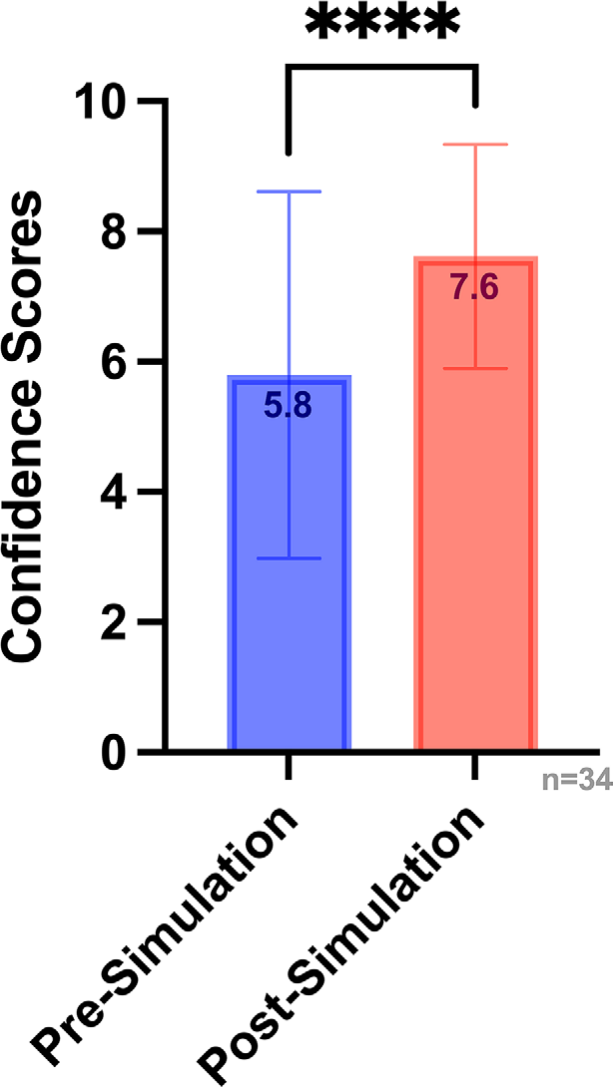
Change in Self‐reported confidence scores. Comparison of pre‐ and post‐simulation confidence scores among participants in the two sessions that included paired data (*n* = 34). Mean confidence increased significantly following the simulation (5.8–7.6; ****p < 0.0001, paired *t*‐test). Error bars represent standard deviation. [Color figure can be viewed in the online issue, which is available at www.laryngoscope.com.]

Findings from the post‐simulation questionnaire demonstrated marked improvements in provider confidence, anxiety reduction, and perceptions of its impact on patient care. Results showed 72% of participants reported increased confidence in managing airway emergencies compared to their pre‐simulation confidence, whereas 86% noted decreased anxiety when faced with future emergencies (*n* = 63). All participants indicated that the simulation would have a positive impact on patient care and safety, and 93% felt that the in situ format was valuable (Figure 2). Among the four simulation sessions, two included paired pre‐ and post‐simulation confidence scores. In these sessions, there was a statistically significant increase in confidence following the simulation (mean difference 1.82 ± 1.51, *t*(33) = 7.06, *p* < 0.0001), with average confidence scores rising from 5.8 to 7.6 on a 10‐point scale (Figure 3).

Qualitative feedback from the debrief sessions highlighted improvements in comfort with airway management equipment. Participants reported that using equipment directly from the airway cart in its standard clinical storage location allowed them to familiarize themselves with available resources and troubleshoot potential challenges in real time.

Improvements in interdisciplinary teamwork and communication were also frequently noted in post‐simulation reflections. Of the 36 written responses collected from the third simulation session, 11 participants referenced improved communication as a key takeaway, and 9 cited increased familiarity with identifying and using airway management equipment. In total, 56% of respondents independently identified these as key learning points.

Across all simulations, participants described the in situ format as beneficial for reinforcing team‐based coordination and equipment utilization, with reflections emphasizing the importance of practicing both technical and nontechnical skills necessary for airway emergency management.

## 4 Discussion

The in situ airway emergency drill demonstrated measurable improvements in provider confidence, anxiety reduction, and team coordination. Participants identified hands‐on experience with equipment and interdisciplinary communication as key factors in enhancing their preparedness. The ability to train in the clinical setting, rather than a simulation center, allowed teams to refine workflows under realistic conditions.

After the simulation, a detailed review of new emergency equipment helped ensure that all ARRT members were familiar with resources available. This allowed providers to subsequently apply this knowledge in real‐world settings, further reinforcing the importance of integrating resource familiarization into training.

Unlike traditional simulations where team roles are assumed by a single specialty—such as a resident playing the role of a pharmacist—this model engaged the actual multidisciplinary response team. Participants emphasized that working alongside their usual colleagues strengthened role clarity, streamlined decision‐making, and improved coordination under pressure.

In the literature, simulations to enhance staff preparedness for adult airway emergencies have shown promising outcomes but have been separated from the inpatient floor, instead taking place in emergency departments, operating rooms, or dedicated simulation centers [10]. Yang et al. [11] notably performed a multi‐institutional study to assess latent safety threats before and after implementing an in situ simulation for COVID‐19 airway management in the emergency department, demonstrating marked improvements in safety. Knight et al. [12] also showed significant improvements in provider confidence using their emergency airway simulation drill; however, their simulation took place in a center designed to mimic an intensive care unit. Our work is a novel addition to the literature, demonstrating the benefits of emergency airway simulation by incorporating providers' perspectives and taking place in situ on the inpatient floor.

A limitation of this study is a relatively modest sample size, though continued adoption of this training model will provide further data. Additionally, while the in situ approach enhanced realism, debrief sessions identified opportunities for refining scenario design and performance assessments. Standardized outcome measures could also improve future evaluations of training efficacy. Importantly, improvements in performance and overall competency should be objectively assessed to further investigate the benefits of this model.

The feasibility of this model depends on institutional resources, including a dedicated airway response team and simulation infrastructure [13]. Adapting it to different clinical environments may require modifications to accommodate available personnel and equipment, and future studies should assess long‐term skill retention and the impact on clinical outcomes. This work would ideally assess retention at set intervals over multiple months using tests of knowledge and practical skills checklists. To evaluate clinical outcomes in the future, investigators could track time to airway securement, complication rates, and frequency of escalation to surgical airways before and after implementation of the training. Additionally, multi‐center studies could evaluate this training model in settings without dedicated airway response teams to better understand the conditions necessary for successful implementation.

## 5 Conclusion

In situ airway emergency drills are an effective training tool for otolaryngologists and interprofessional collaborators, helping improve teamwork and provider confidence. This approach allows staff to practice technical skills and refine communication in a controlled environment that is more realistic than a siloed simulation center.

Multidisciplinary airway response teams across hospital systems can benefit from in situ simulations tailored to their clinical environments and protocols. Drills conducted in the same spaces where emergencies occur help team members become more familiar with their roles and equipment, which is particularly important for teams whose members span multiple departments. Future studies should focus on evaluating the long‐term impact of in situ drills on patient morbidity and mortality, which would help guide strategies to improve clinical outcomes.

## Disclosure

Meeting: Poster presented at The Triological Society Combined Sections Meeting January 23–25, 2025 in Orlando, FL.

## Conflicts of Interest

The authors declare no conflicts of interest.

## Data Availability

The data that support the findings of this study are available from the corresponding author upon reasonable request.
